# Stereotactic body radiotherapy in early-stage hepatocellular carcinoma: a systematic review and meta-analysis

**DOI:** 10.1016/j.esmogo.2025.100281

**Published:** 2026-01-14

**Authors:** J.K. van Vulpen, M.C. Verwijs, F.K. Gommers, R.A.H. van Eijck van Heslinga, S. van Meer, C.J.R. Verstraete, J. Hagendoorn, M.G.E.H. Lam, N. Haj Mohammad, M.L.J. Smits, M.N.G.J.A. Braat, M.P.W. Intven, J. de Bruijne

**Affiliations:** 1 Department of Radiation Oncology, University Medical Center Utrecht, Utrecht University, Utrecht, The Netherlands; 2 Departments of Gastroenterology and Hepatology, University Medical Center Utrecht, Utrecht University, Utrecht, The Netherlands; 3 Departments of Surgical Oncology, University Medical Center Utrecht, Utrecht University, Utrecht, The Netherlands; 4 Departments of Radiology and Nuclear Medicine, University Medical Center Utrecht, Utrecht University, Utrecht, The Netherlands; 5 Departments of Medical Oncology, University Medical Center Utrecht, Utrecht University, Utrecht, The Netherlands

**Keywords:** radiotherapy, primary liver cancer, ablative treatment, local control, toxicity

## Abstract

**Background:**

First-line treatment strategies for (very) early-stage hepatocellular carcinoma (HCC) include liver transplantation, surgical resection and thermal ablation. Stereotactic body radiotherapy (SBRT) has recently been included in the European Association for the Study of the Liver Clinical Practice Guidelines on the management of hepatocellular carcinoma as an alternative ablative strategy. We aimed to carry out a systematic review and meta-analysis on oncological outcomes and toxicity of SBRT focused on early-stage HCC treatment.

**Materials and methods:**

We carried out a systematic literature search in PubMed, Embase, and the Cochrane Library from inception throughout October 2022. Studies of SBRT targeting treatment-naive (very) early-stage HCC (BCLC 0/A) patients were included.

**Results:**

One prospective and 15 retrospective studies were included in this review. In aggregate, SBRT in 1249 patients resulted in a 1-, 2-, and 3-year local control rate of 94% (95% CI 92%-97%), 89% (95% CI 85%-93%), and 79% (95% CI 68%-90%), respectively. The pooled results of the 1-, 2-, and 3-year overall survival rate were 90% (95% CI 85%-94%), 75% (95% CI 63%-87%), and 59% (95% CI 45%-73%), respectively. Grade ≥3 toxicity was observed in 2% of patients (95% CI 0%-4%).

**Conclusion:**

This systematic review and meta-analysis showed that SBRT is an effective and safe treatment modality for treatment-naive patients with early-stage HCC. The data support incorporation of SBRT as a treatment option in the treatment algorithms for (very) early-stage HCC.

## Highlights

• SBRT is emerging as a non-invasive ablative treatment option for early-stage HCC.
• With SBRT, high radiation doses can be successfully delivered to focal tumours.
• Good local control rates indicate the effectiveness of SBRT for treatment of early-stage HCC.
• The favourable toxicity profile of SBRT underlines its role as a safe alternative treatment option.

## Introduction

Primary liver cancer is the sixth most common cancer type and third leading cause of cancer-related deaths globally. Hepatocellular carcinoma (HCC) accounts for ∼75%-85% of all primary liver cancers.^1^ Adequate management of HCC is highly dependent on accurate prognostic assessment and treatment allocation. The Barcelona Clinic Liver Cancer (BCLC) classification model^2^ is a widely used staging system and is incorporated into the ‘Management of hepatocellular carcinoma guideline’ by the European Association for the Study of the Liver Clinical Practice Guidelines committee.^3^

For (very) early-stage HCC (BCLC-0 and BCLC-A), first-line treatment strategies include liver transplantation, ablation, and surgical resection.^2^ Stereotactic body radiotherapy (SBRT), a specialized type of external beam radiotherapy (EBRT), has gained interest as an additional treatment approach for early-stage HCC because of its non-invasiveness in combination with promising local control rates and overall survival rates. This technique allows highly precise delivery of high doses of radiation to small tumours. Although EBRT is often applied in dismal clinical condition (i.e. patients are not eligible for surgery or ablation, have a more advanced Child–Pugh classification and/or have worse performance status), several meta-analyses including both retrospective and prospective studies have demonstrated the safety and efficacy of EBRT for all BCLC stages, including SBRT and proton beam therapy.^4^, ^5^, ^6^, ^7^ These meta-analyses cover a broad range of tumour stages and often include pretreated patients, complicating evaluation of the isolated effect of SBRT for HCC. In the recent European Association for the Study of the Liver (EASL) Clinical Practice Guidelines and 2025 BCLC update, SBRT has been incorporated as an alternative ablative modality to percutaneous ablation for well-compensated patients with solitary tumours in cases where the lesion is not amenable to ablation or the patient is deemed non-operable or ineligible for transplantation.^3^^,^^8^ To further complement the evidence for SBRT as first-line treatment, the current systematic review and meta-analysis aim to provide an overview on the oncological outcomes and toxicity or safety of SBRT, specifically focused on treatment-naive, early-stage HCCs.

## Material and Methods

This systematic review and meta-analysis was conducted in accordance with the PRISMA (Preferred Reporting Items for Systematic reviews and Meta-Analyses) guidelines (Supplementary Appendix S1, available at https://doi.org/10.1016/j.esmogo.2025.100281).

### Literature search

A systematic literature search was conducted in PubMed, Embase, and the Cochrane Library in October 2022, using a combination of the following search terms and their synonyms: ‘stereotactic body radiotherapy’ and ‘hepatocellular carcinoma’. Duplicate titles were excluded and the title and abstract of the remaining papers were examined using predefined inclusion criteria.

### Study inclusion criteria

The criteria for inclusion in our study were: (i) at least 10 SBRT-treated patients with primary, early-stage HCC lesions; (ii) the utilization of SBRT (definition according to Guckenberger et al.^9^) as a primary treatment; (iii) with curative intent; (iv) reported outcomes in either overall survival (OS), local control, or toxicity. The exclusion criteria were as follows: (i) patients treated with ablation, surgical resection, transarterial chemo-/radio-embolization, systemic therapy or radiotherapy before SBRT; (ii) ≥10% BCLC B, C, or D cases on study level; (iii) >50% of included cases with macrovascular invasion or portal vein tumour thrombosis; (iv) SBRT intentionally planned as a bridge to transplant; (v) conference abstracts and reviews. When multiple studies from a single institution involved overlapping patients, the study with the largest proportion of patients diagnosed with BCLC stage 0/A was included.

### Selection of studies and data extraction

Titles and abstracts of all retrieved articles were screened. Full-text articles of potentially relevant papers were assessed independently for eligibility by two investigators. Relevant data were collected using a predefined data extraction form and any disagreements were resolved by discussion and consultation of a third expert. The collected study data was related to (i) study characteristics (authors, year of publication, study type, study size), (ii) participant characteristics (age, sex, tumour size, proportion of single lesions, Child–Pugh classification, and viral aetiology), (iii) treatment information (total irradiation dose, fractionation scheme, and follow-up period), and (iv) outcomes. The primary outcomes were 1-year local control and 1-year overall survival. Local control was evaluated by using (contrast-enhanced) CT or magnetic resonance imaging and was defined as freedom from local disease progression according to the RECIST v1.1 or modified RECIST guidelines,^10^, ^11^, ^12^, ^13^, ^14^, ^15^, ^16^, ^17^, ^18^, ^19^, ^20^ according to EASL criteria,^21^ according to World Health Organisation criteria,^22^ or not specified.^23^, ^24^, ^25^ The secondary outcomes were 2- and 3-year local control and overall survival, and incidence of any acute or late Common Terminology Criteria for Adverse Events ≥grade 3. Toxicities rated as grade 3 are severe or medically significant, but not immediately life threatening; grade 4 include life-threatening consequences; and grade 5 death due to radiotherapy-related adverse events. In case of unavailable numerical data, estimations of the survival or local control rate were derived from the descriptive graphs if possible (one study).

### Quality assessment

The methodological quality on outcome level for each study was assessed by using a modified version of the Joanna Briggs Institute Critical Appraisal of Cohort Studies tool.^26^ As no control group was incorporated into the studies included in this systematic review, the initial three questions of the standardized tool (focusing on comparison between two groups) were omitted. Studies with a score of ≥75% were categorized as high quality, between 50% and 74% as medium quality, and <50% as low quality. Given the retrospective design of 15/16 included studies, and the non-controlled setting, there is an inherent high susceptibility to bias associated with the included set of studies. Application of the Joanna Briggs Institute tool further classified the quality of the individual studies into high quality (nine studies) and medium quality (seven studies). No studies were excluded from the review based on the quality assessment. The detailed quality assessment is presented in Supplementary Appendix S1, available at https://doi.org/10.1016/j.esmogo.2025.100281.

### Data synthesis and statistical analysis

Statistical analyses were conducted using OpenMeta[Analyst]. Weighted random-effect meta-analyses were used to evaluate primary and secondary endpoints. Results for the different outcomes were presented using forest plots. Heterogeneity was assessed using the I^2^ statistic and Cochran Q test. Significant heterogeneity was considered to be present if the I^2^ statistic was >50%, and the *P* value of the Q test was <0.10.

### Ethics

The study is based exclusively on published literature.

## Results

### Characteristics of included studies

**Figure 1:**
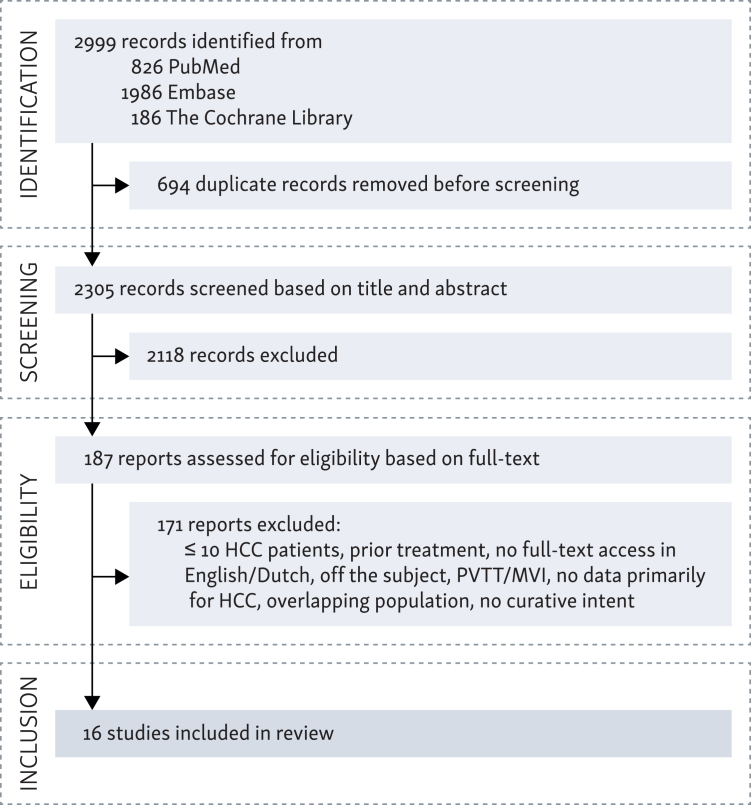
**The study identification process.** Vertical boxes indicate each stage of the screening, horizontal boxes present more detailed information about the process, including the steps carried out in each stage.

The search in the PubMed, Embase, and Cochrane databases identified 2999 studies. After the exclusion of duplicate studies, 2305 studies were screened based on title and abstract. The remaining 187 studies were assessed for eligibility based on full text. The final study selection consisted of 16 studies. The study identification process is shown in Figure 1, based on the PRISMA 2020 flow diagram.

The study period ranged from 2004 to 2020. A retrospective design was employed in 15 out of 16 studies, accounting for 94% of the total. The total number of patients receiving SBRT as a primary treatment of HCC was 1249. The median age of the patients varied between 53 and 77 years. The majority of participants were male. The majority of patients had cirrhosis, with a median proportion of patients with a Child–Pugh A score of 84% (range 57%-100%). The median percentage of hepatitis B virus and hepatitis C virus as aetiology for cirrhosis among studies was 73% (range 8%-100%), and 11% (range 0%-66%), respectively. The majority of patients presented with a single lesion (95%-100%), with median tumour diameters ranging from 1.7 cm to 3.8 cm. A detailed overview of the study specific information is provided in Table 1.

**Table 1:** Study details of SBRT in patients with early HCC

Authors (year)	Type	Study period	*N*	Single lesion, in %	Median age (range), in years	Male, in %	Median tumour diameter (range), in cm	CP A/B, in %	HBV/HCV, in %	Reported outcomes
Andolino et al. (2011)^10^	P	2005-2009	37	95	63 (24-85)	81	3.5 (1.0-6.5)	64/35	8/43	2-year LC; 1-, 2-, 3-year OS
Chen et al. (2020)^11^^a^	R	2011-2019	28	96	A: 61 (42-80)	82	A: 1.7 (1.0-4.5)	96/4	86/11	1-, 3-year OS
					B: 75 (41-83)		B: 2.9 (0.9-4.3)			
Dewas et al. (2012)^12^	R	2007-2010	42	NR^b^	69 (43-85)	76	47.5 (1.4-499)^c^	86/14	NR	1-, 2-year LC, 1-, 2-year OS
Han et al. (2022)^13^	R	2011-2020	240	100	54 ± 9.4^d^	75	2.1 (1.5-3.03)^e^	91/9	100/0	1-, 2-, 3-year LC
Hanazawa et al. (2017)^14^	R	2012-2015	17	100	77 (63-85)	77	7.9 (3.9-51.0)^c^	82/18	NR	1-, 2-, 3-year LC, 1-, 2-, 3-year OS
Janoray et al. (2014)^15^	R	2010-2012	21	NR^b^	71 (56-81)	62	2.3 (1.4-6.0)	57/19	NR	1-year LC
Lai et al. (2020)^19^	R	2009-2016	72	100	57 (30-84)	85	3.4 (1.5-5.0)	88/13	100/0	1-, 2-, 3-year OS
Liu et al. (2020)^18^^f^	R	2013-2018	59	NR	66 (35-88)	85	3.8 (1.5-17 cm)	88/12	9/40	1-, 2-year LC, 1-, 2-year OS
Mendiratta et al. (2020)^17^	R	2008-2016	45	NR	65 (22-90)	80	3.0 (0.9-10.5)^g^	71/29	9/47	1-, 2-, 3-year LC, 1-, 2-, 3-year OS
Nouhaud et al. (2013)^21^^h^	R	2007-2012	14	65	73 (50-85)	95	2.4 (0.5-9.8)	100/0	NR	1-year LC, 1-, 2-year OS
Rajyaguru et al. (2018)^24^^i^	R	2004-2013	296	NR	4.1%: ≤49 26.0%: 50-59 28.4%: 60-70 41.6%: ≥71	70	15.5%: <2 33.1%: 2-3 26.4%: 3-4 25.0%: 4-5	NR	NR	1-, 2-, 3-year OS
Shin et al. (2022)^16^	R	2011-2017	34	100	63 ± 11^d^	65	1.41 ± 0.42^d^	NR	59/9	1-, 3-year LC, 1-, 2-, 3-year OS
Shiozawa et al. (2015)^23^	R	2011-2014	35	100	75 (55-89)	69	2.9 ± 1.2^d^	80/20	11/66	1-, 2-year LC, 1-year OS
Su et al. (2020)^20^	R	2009-2017	167	NR	56 (47, 65)^e^	84	3.4 (2.4-5.2)	82/18	87/NR	1-, 2-, 3-year LC, 1-, 2-, 3-year OS
Sun et al. (2020)^25^	R	2011-2015	122	100	54 ± 9.4^d^	74	2.6 ± 1.1^d^	91/9	93/7	1-, 2-, 3-year LC, 1-, 2-, 3-year OS
Yang et al. (2010)^22^	R	2004-2007	20	100	53 (43-68)	75	3.2 (2.1-4.8)	60/40	NR	1-, 2-, 3-year OS

CP A, Child–Pugh class A; CP B, Child–Pugh class B; HBV, hepatitis B virus; HCV, hepatitis C virus; LC, local control; NR, not reported; OS, overall survival; P, prospective cohort study; R, retrospective study.

a Includes patients who were eligible for surgery but refused (group A, 39%) and patients for whom surgery or RFA were impractical due to technical or medical constraints (group B, 61%).

b Maximum of two lesions per patient.

c Median gross tumour volume, in cm^3^.

d Mean ± SD.

e Median (IQR).

f Baseline characteristics pertain to the entire cohort, of which the BCLC 0/A subgroup accounts for 61%.

g Mean tumour diameter (range).

h Baseline characteristics pertain to the entire cohort, of which the BCLC 0/A subgroup accounts for 70%.

i Data regarding age and tumour diameter exclusively available as subgroup-specific proportions.

Detailed radiation treatments characteristics of the selected studies are summarized in Table 2. The total irradiation dose ranged from 21 to 60 Gy delivered in 1-10 fractions. The median follow-up varied between 13 and 67 months.

**Table 2:** Radiation treatment characteristics

Authors (year)	Median total dose (range), in Gy	Median number of fractions (range)	Median follow-up (range), in months
Andolino et al. (2011)^10^	48 (40-48)	3 (3-5)	27 (2-52)
Chen et al. (2020)^11^	(48-60)	(5-10)	23 (4-99)
Dewas et al. (2012)^12^	45 (27-45)	3 (2-3)	15 (12-17)^a^
Han et al. (2022)^13^	(48-54)	(5-8)	30
Hanazawa et al. (2017)^14^	50 (45-50)	5 (5-10)	16
Janoray et al. (2014)^15^	45 (45-60)	3	10 (1-20)
Lai et al. (2020)^19^	(36-48)	(3-5)	67 (8-126)
Liu et al. (2020)^18^	86 (48-113)^b^	3 or 5	13 (3-65)
Mendiratta et al. (2020)^17^	21-60	(3-5)	>24
Nouhaud et al. (2013)^21^	60 (36-60)	NR	24 (16-30)
Rajyaguru et al. (2018)^24^^c^	9%: <30 13%: 30-39 42%: 40-49 22%: ≥50 14%: unknown/missing	9%: 1-2 80%: 3-5 6%: ≥6 5%: unknown/missing	25 (14-41)
Shin et al. (2022)^16^	55 (40-60)	(3-5)	NR
Shiozawa et al. (2015)^23^	60	(3-5)	12 (7-35)
Su et al. (2020)^20^	28-50	1-5	35
Sun et al. (2020)^25^	48-54	5-8	60 (4-117)
Yang et al. (2010)^22^	50	10	35 (11-44)

NR, not reported.

a Median (95% CI).

b Median biologically effective dose with tumour α/β ratio of 10 Gy (range), in Gy.

c Data regarding total dose and number of fractions exclusively available as subgroup-specific proportions.

### Oncological outcomes

**Figure 2:**
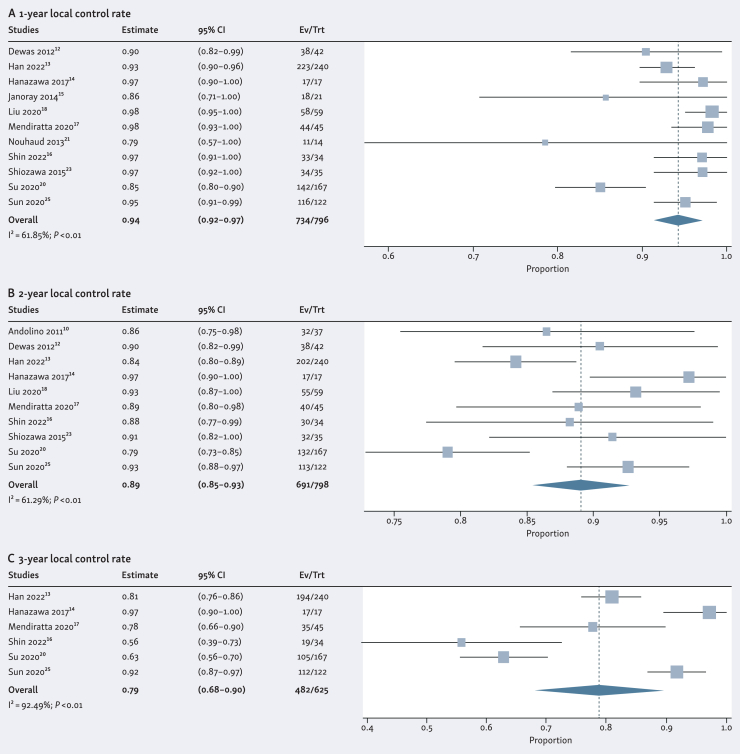
**Forest plots of the (A) 1-year, (B) 2-year, and (C) 3-year local control rate**. Proportions for each trial are represented by a square and the horizontal line crossing the squares indicates the 95% confidence interval. The diamonds represent the estimated overall effect of the meta-analysis based on a random effect mod

**Figure 3:**
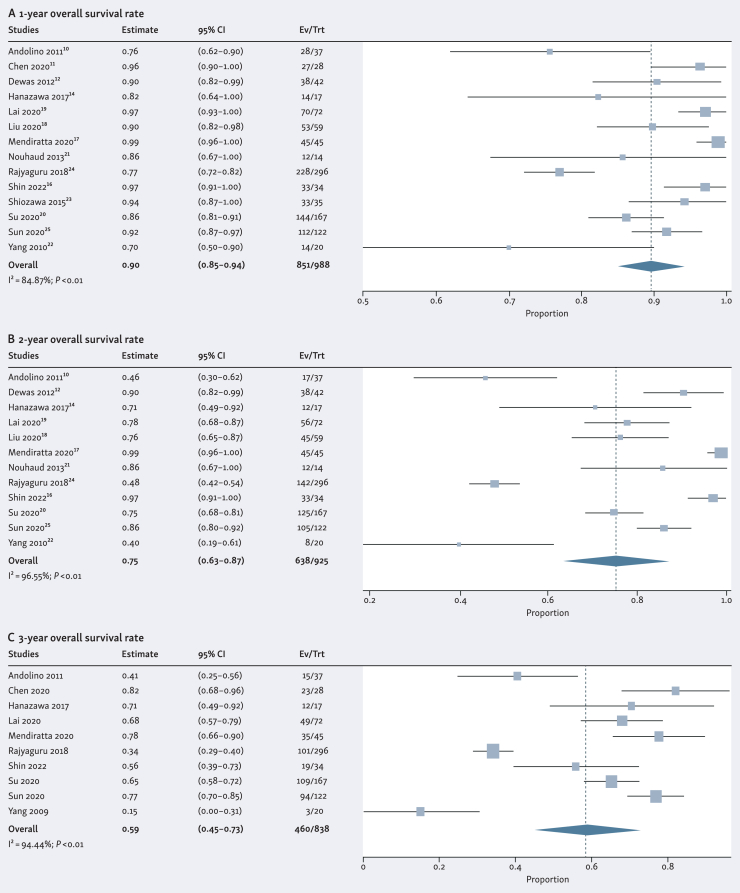
**Forest plots of the (A) 1-year, (B) 2-year, and (C) 3-year overall survival rate**. Proportions for each trial are represented by a square and the horizontal line crossing the squares indicates the 95% confidence interval. The diamonds represent the estimated overall effect of the meta-analysis based on a random effect model.

Meta-analyses of the oncological outcomes are visualized as forest plots (Figures 2 and 3). Applying a random effect model, the pooled results of the 1-, 2-, and 3-year local control rate demonstrated 94% (95% CI 92%-97%), 89% (95% CI 85%-93%) and 79% (95% CI 68%-90%), respectively. The pooled results of the 1-, 2-, and 3-year overall survival rate were 90% (95% CI 85%-94%), 75% (95% CI 63%-87%) and 59% (95% CI 45%-73%), respectively. A summary of the pooled results is presented in Table 3.

**Table 3:** The pooled rates of local control, overall survival, and ≥grade 3 toxicity

	Local control			Overall survival			≥Grade 3 toxicity
	**1-year**	**2-year**	**3-year**	**1-year**	**2-year**	**3-year**	
*n*	796	798	625	988	925	838	257
*P* value for heterogeneity	<0.01	<0.01	<0.01	<0.01	<0.01	<0.01	0.31
I^2^	61.85	61.29	92.49	84.87	96.55	94.44	16.34
Pooled result, %	94	89	79	90	75	59	2
95% CI, %	92-97	85-93	68-90	85-94	63-87	45-73	0-4

CI, confidence interval; I^2^, the Higgins I^2^ index; *P* value for heterogeneity, corresponding to the Cochran's Q test; Random effects model, via the DerSimonian–Laird method.

### Treatment-related toxicity

**Figure 4:**
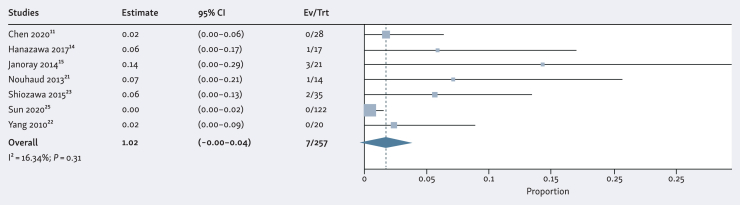
**Forest plot of the ≥ grade 3 toxicity.** Proportions for each trial are represented by a square and the horizontal line crossing the squares indicates the 95% confidence interval. The diamond represents the estimated overall effect of the meta-analysis based on a random effect model.

Figure 4 shows a forest plot depicting late grade 3-5 toxicity after SBRT for BCLC-0/A HCC. Toxicity results were reported in seven studies (*n* = 257 cases). The pooled proportion of ≥grade 3 toxicity was 2% (95% CI 0%-4%). The most frequently reported ≥grade 3 adverse events were isolated elevated liver enzymes. In 4 out of 257 cases, grade 5 toxicity occurred: 3 patients (1%) developed fatal radiation induced liver disease (RILD) and 1 patient (0.4%) died of gastric bleeding.

## Discussion

This systematic review and meta-analysis aimed to assess the oncological outcomes and toxicity profile of SBRT for patients with treatment-naive, (very) early-stage HCC. This meta-analysis, comprising 16 observational studies and 1249 patients with HCC, demonstrated a pooled local control rate of 94% and a high 1-year overall survival of 90%, accompanied by low toxicity.

Previous meta-analyses on SBRT effects for HCC showed comparable favourable pooled 1-year (85.7%-96.0%) and 3-year (83.9%-91.0%) local control rates.^4^, ^5^, ^6^, ^7^ A recent meta-analysis focusing specifically on longer term outcome of SBRT in all types of HCC also demonstrated a durable long-term local control of 82% after 5 years. The clinical practice guidelines that were subsequently developed on behalf of the International Stereotactic Radiosurgery Society recommend that SBRT can be considered in HCC <3 cm with favourable local control and survival outcomes, and that durable long-term local control is still to be expected for HCC ≥3 cm.^7^ The observed overall survival rates in our meta-analysis (90% and 59% at 1-year and 3-year, respectively) are lower than those reported after resection (1-year 93.1%-98.3% and 3-year 67.2%-92.2%) and radiofrequency ablation (1-year 87.0%-96.0% and 3-year 69.6%-82.3%) for (very) early-stage HCC.^27^, ^28^, ^29^, ^30^ Results of comparative effectiveness studies for SBRT versus thermal ablation showed inconsistent results: Wahl et al. reported similar 2-year local progression-free survival rates for SBRT (83.3%) and radiofrequency ablation (RFA) (80.2%), with no significant difference in OS.^31^ In contrast, Rajyaguru et al. observed superior 5-year OS with RFA compared with SBRT (29.8% versus 19.3%),^24^ whereas Kim et al. found that SBRT was associated with a lower risk of local recurrence but a higher 2-year mortality rate (25.7% versus 18.9%).^32^ The inconsistent findings are likely due to differences in patient populations, including variations in disease stage and underlying aetiology of liver disease. More recently, two randomised controlled trials on RFA versus radiotherapy (one SBRT and one proton radiotherapy) for small HCC were published, showing that radiotherapy achieved superior local progression-free survival with comparable OS.^33^^,^^34^

It is important, though, to note that until recently, SBRT has not been a first-line treatment option for HCC patients, and consequently SBRT was mainly applied in inoperable patients due to comorbidities, when resection and ablation were technically not feasible or due to patient preferences. For example, Dewas et al. mentioned that SBRT was considered for patients for whom standard treatments were not feasible,^12^ and Mendiratta et al. included patients with HCC who were difficult to assess percutaneously or patients with contraindications to other forms of locoregional therapy.^17^ As a consequence, the selected patient population might explain the somewhat lower local control and overall survival rates for SBRT-treated patients. Indeed, previous reports indicate that risk factors for not receiving first-line HCC treatment options such as ablation or resection include portal hypertension, older age (>65 years), and presence of comorbidities.^35^^,^^36^ Interestingly, real-world data indicate that a substantial proportion of patients with early-stage HCC do not receive surgery or ablation,^37^, ^38^, ^39^ underscoring the need to incorporate alternative treatment options into therapeutic algorithms and (international) guidelines for well-compensated patients.

Importantly, EBRT has made significant technological developments over the past years. Whereas the ability to deliver high, adequate doses for HCC with conventional EBRT was previously limited, nowadays tumoricidal doses can be safely and precisely delivered by SBRT with millimetre-level accuracy. Moreover, by using methods to control for respiratory-induced liver motion (i.e. tracking and gating), the volume of irradiated healthy liver and adjacent bowel can be even further reduced.^40^ In the meantime, magnetic resonance imaging-guided SBRT is increasingly being used to enable optimized visualisation of tumours, taking away the need for implantation of gold markers/fiducials. Also, functional liver imaging is being incorporated into SBRT planning, aiming at further preserving the volume of functional liver remnant.^40^ Of note, this meta-analysis included both recent studies and studies from the earlier days of liver treatment. As SBRT has evolved largely over the years, current outcomes might even be better than the pooled estimates presented in the meta-analysis.

Also, in the studies included in the current review, different techniques (i.e. tracking, gating, and free-breathing) were used, possibly affecting clinical outcomes due to dosimetric differences. Because of multiple varying parameters (e.g. techniques, radiation doses, irradiated volumes) between studies however, no valid comparison for technique effect on the clinical outcome can be made.

In the past few years, there have been major advances in the armamentarium for treatment of HCC, leading to updates in treatment algorithms for HCC management. In the most recent evidence-based practice guidelines from the American Society for Radiation Oncology, a strong recommendation was made for the use of EBRT as a potential first-line treatment option in patients with liver-confined HCC who are not candidates for surgery or ablation.^41^ Furthermore, several other clinical practice guidelines (e.g. from the National Comprehensive Cancer Network,^42^ European Society for Medical Oncology,^43^ and American Association for the Study of Liver Diseases^44^) have incorporated external beam radiotherapy as an alternative treatment option in their treatment algorithms. Moreover, in the most recent update of the EASL Clinical Practice Guidelines on the management of HCC, EBRT now is included as an alternative to percutaneous ablation for single tumours within Milan criteria unsuitable for resection or transplantation, when there is a significant risk of post-ablation recurrence based on size (>3 cm) or location (e.g. in contact with large vessels). In their paper, the committee highlights the need for a multiparametric assessment of individual risks and benefits, considering the patient's perspective, by a multidisciplinary team encompassing various specialties.^3^ Subsequently, in the recent 2025 BCLC update, EBRT was considered to have encouraging outcomes in early disease, and has been incorporated as an ablative option in BCLC-0 patients.^8^

In addition to the good local control rate, other major advantages of SBRT are its non-invasiveness and largely favourable toxicity profile in non-cirrhotic patients or patients with well-compensated cirrhosis. Traditionally, RILD has been a frequently cited concern in the radiotherapeutic management of HCC. The incidence of RILD has strongly decreased as a result of advancements in image-guided adaptive techniques resulting in minimal damage to non-tumorous liver parenchyma, however.^45^ This meta-analysis provides support for minimal severe toxicity after SBRT, as the pooled proportion of ≥grade 3 toxicity was merely 2%. Moreover, the non-invasive nature of the procedure allows patients to receive treatment in outpatient clinics. Finally, SBRT has the possibility to treat tumours unsuitable for thermal ablation, in particular those adjacent to major blood vessels, bile ducts, or the diaphragm.^46^

This systematic review and meta-analysis have several limitations that should be acknowledged. Firstly, we included a limited number of studies with a small patient population. Secondly, the majority of included studies had an observational, retrospective nature, which comes with an inherent high susceptibility to bias, especially for the toxicity outcome. Also, unfortunately only a limited number of studies reported on toxicity, so our pooled results should only be regarded as an indication of the true toxicity rate. Furthermore, not all clinical baseline information that may be of interest was available. For example, some studies lacked baseline data on performance status, underlying aetiology of HCC, number of lesions, and stage of disease. To a small extent, there may therefore be contamination from more advanced stage disease patients with inferior prognosis. We hypothesize that BCLC 0/A patients might in fact have slightly better outcome than the pooled estimates of our meta-analysis indicate. Secondly, there were not sufficient data on progression-free survival or requirements of additional treatments after SBRT over time to include those outcomes in the current meta-analysis. This emphasizes the need for further prospective studies, with strict registration of these outcomes. Thirdly, most included studies were carried out in Asian countries with high risk of HCC, in which there is another distribution of risk factors (i.e. HBV/HCV prevalence) compared with other, low-risk countries (e.g. Northern Europe, USA), which may affect generalisability. Finally, despite the stringent inclusion criteria used in the search strategy, there was still considerable heterogeneity between the included studies. A strength of this study over several other systematic reviews was the tailored research question, providing an oversight of evidence on primary SBRT treatment of early-stage (BCLC 0/A) HCC, specifically.

### Conclusions

In conclusion, results of this systematic review and meta-analysis provide additional evidence supporting the feasibility, efficacy, and safety of SBRT as a primary treatment of (very) early-stage HCC when ablation or resection is not feasible or preferred. Given its high local control rates and favourable toxicity profile, SBRT represents an effective and non-invasive alternative for BCLC 0/A HCC patients, supporting its integration into HCC treatment algorithms.
