# Supplementary material for: Stereotactic body radiotherapy in early-stage hepatocellular carcinoma: a systematic review and meta-analysis
Source: ESMO Gastrointest Oncol. 2026 Jan 14;11:100281. doi: 10.1016/j.esmogo.2025.100281 (PMC13080873; doi:10.1016/j.esmogo.2025.100281)
Supplement: Supplemental Appendix [file mmc1.docx]

**Supplementary Appendix S1: The quality assessment based on the JBI Critical Appraisal of Cohort Studies tool**

| Author | Year | Q4^a^ | Q5^b^ | Q6^c^ | Q7^d^ | Q8^e^ | Q9^f^ | Q10^g^ | Q11^h^ | Y/Total | Score (%) |
| --- | --- | --- | --- | --- | --- | --- | --- | --- | --- | --- | --- |
| Andolino | 2021 | Y | Y | Y | Y | Y | N | Y | Y | 7/8 | 88% |
| Chen | 2020 | Y | Y | Y | Y | Y | N | Y | N | 6/8 | 75% |
| Dewas | 2012 | Y | Y | Y | Y | U | U | Y | Y | 6/8 | 75% |
| Han | 2022 | Y | Y | Y | Y | Y | U | Y | Y | 7/8 | 88% |
| Hanazawa | 2017 | N | N | Y | Y | U | U | Y | Y | 4/8 | 50% |
| Janoray | 2014 | N | N | Y | Y | U | U | Y | Y | 4/8 | 50% |
| Lai | 2020 | Y | Y | Y | Y | Y | U | Y | Y | 7/8 | 88% |
| Liu | 2020 | N | N | Y | Y | U | U | Y | Y | 4/8 | 50% |
| Mendiratta | 2020 | N | N | Y | Y | Y | N | Y | Y | 5/8 | 63% |
| Nouhaud | 2013 | N | N | Y | Y | Y | N | Y | Y | 5/8 | 63% |
| Rajyaguru | 2018 | Y | Y | Y | Y | Y | Y | Y | Y | 8/8 | 100% |
| Shin | 2022 | Y | Y | Y | Y | U | U | Y | Y | 6/8 | 75% |
| Shiozawa | 2015 | N | N | Y | Y | U | U | Y | Y | 4/8 | 50% |
| Su | 2020 | Y | Y | Y | Y | Y | N | Y | Y | 7/8 | 88% |
| Sun | 2020 | Y | Y | Y | Y | Y | U | Y | Y | 7/8 | 88% |
| Yang | 2009 | N | N | Y | Y | Y | N | Y | Y | 5/8 | 63% |

Studies with a score of 75% or higher were categorized as high quality, within 50% and 74% as medium quality, and less than 50% as low quality. Abbreviations: Q: question (*see below)*; Y: Yes; N: No; U: Unknown.

| ^a^ | Were confounding factors identified? |
| --- | --- |
| ^b^ | Were strategies to deal with confounding factors stated? |
| ^c^ | Were the groups/participants free of the outcome at the start of the study (or at the moment of exposure)? |
| ^d^ | Were the outcomes measured in a valid and reliable way? |
| ^e^ | Was the follow up time reported and sufficient to be long enough for outcomes to occur? |
| ^f^ | Was follow up complete, and if not, were the reasons to loss to follow up described and explored? |
| ^g^ | Were strategies to address incomplete follow up utilized? |
| ^h^ | Was appropriate statistical analysis used? |
